# Annotations

**Published:** 1904-12-17

**Authors:** 


### ANNOTATIONS.

### Sanitation in Brazil.

PERHAPS the very last character which, in this ountry, a South American Republic would be nerally expected to assume would be that of a oneer in sanitary legislation, but it has, neverthe-ss, been undertaken by Brazil. The report made the government of the Brazilian States by Dr. ouvêa, their delegate to the conference on tuber-ulosis held last May at Copenhagen, has just been ublished, and has as an appendix a summary of the laws relating to public health which were proposed by the president, Senhor Rodrigues Alves, on assum-ing the reins of government at the end of last year, nd which were enacted to come into operation last March. They not only provide very completely for he notification of all infectious diseases, including uberculosis, and for proper restrictive measures when notification has been made, but they go so far as to impose the responsibility of notifying, in the absence of a medical attendant or of a relative, or of a landlord, upon "le voisin le plus proche, dès qu'il en aura connaissance ou qu'il soupçonnera la nature infectieuse de la maladie." Certificates of sound sanitary condition are required from all persons who let either houses or lodgings, and the penalties for breach of the law are severe, often commencing at £10 sterling. Persons suffering from certain forms of tuberculosis are forbidden to engage in certain inds of employment; and arrangements are sanc-ioned for combining the forces of trade and of philanthropic societies in endeavours to provide sanitaria and other means for the treatment of the sick. Provision is made for the employment of an efficient sanitary staff; and, if the enforcement of he new laws in practice should be such as to corre-pond with their stringency upon paper, the health f the communities concerned ought to be speedily nd very materially benefited. We may judge of he room which existed for improvement by the fact hat, according to Dr. Gouvêa, the annual mortality from phthisis and other tuberculous diseases, in the city of Rio-de-Janeiro, amounted, two years ago, to 60 per 1,000 of the population.

### An Orthodox Posology.

IN a recent address a professor of therapeutics is reported to have criticised the dosage of the official remedies as this is defined in the pages of the "British Pharmacopœia," and to have ex-pressed a hope that in future the doses would be phrased in such a way as to afford more "guidance to the prescriber." The plan he proposes is that a total or summated dose permissible for a given time (say 12 or 24 hours) should be stated, and that the prescriber should be left to use his discretion regarding the number of parts into which this total should be divided. In other words, the suggestion is that the General Medical Council as the compilers of the Pharmacopœia shall fix for each remedy a standard maximum dose, beyond which the physician may not pass. There is, we venture to think, not the slightest chance of any such suggestion being adopted, but the position and reputation of the author may lend a tran-sitory importance to it. In our judgment, such a proposal argues a complete failure to appreciate the purpose of a national Pharmacopœia, and would fetter the freedom of the physician in a manner wholly antagonistic to the interests of the individual patient and to the advance of the therapeutic art. The very basis of medical practice rests on the assumption that the practitioner in attendance is alone competent to determine the required treatment and it is on him that full responsibility rests. But if an authoritative list of doses is to be issued and supported by Act of Parlia-ment the physician obviously is no longer entirely free or fully responsible. Further, it is an appro-priate question to inquire what special competence the Medical Council possesses for the proposed task. The fact is that the authority of the Pharmacopœia exists not for the standardisation of doses but for the precise definition of the terms used in physicians' prescriptions. The statement of an official dose has some value because it aids the pharmacist in the detection of some chance error and so protects the public. But the profession has no intention of sub-mitting to an official limitation of its freedom by the recognition of an orthodox posology.

### Examiners and Inspectors.

THE recent debates in the General Medical Council have illustrated the delicate nature of the duties undertaken by those authorised by the Council to inspect the various qualifying examinations con-ducted in the several parts of the United Kingdom. The necessity for such inspections will be universally admitted and hence there may be said to be a *primâ facie* case in favour of regarding the official reports with the respect due to judicial deliverances. Cer-tainly in so far as they deal with matters of fact there will be in the general mind but little tendency to go behind them, and in this relationship at all events they may safely be accepted as the observations of impartial and expert onlookers. But when the field of opinion is entered it must be remembered that there are many questions associated with examina-tions and the manner in which these are best con-ducted that excite strong differences of opinion among men equal to one another both in honesty and experience. Indeed this fact is one of the strongest reasons for the regular inspection of the examinations by men from different schools and familiar with various methods. Hence criticism arises, out of which it may be hoped a general improvement in the standard and plan of medical examinations will emerge. It is therefore to be regretted that the report on the examinations of the University of Edinburgh should have excited in the representative of that University so keen a note of resentment. It may be allowed that the criticism advanced by the Medical Council's inspectors was somewhat deficient in precision and in definiteness of application. But to denounce it as an attack on the "honour" of the University was surely an excessive statement hardly calculated to excite sympathy for the defence. Whatever temporary friction may arise from the procedure of inspection the general advantage is a considerable one, and this applies usually in a special measure to the authorities who find themselves the subjects of criticism.

